# The Paris system for reporting urinary cytology: what worked and what still needs to be improved

**DOI:** 10.1111/his.70038

**Published:** 2025-12-12

**Authors:** Eva M Wojcik

**Affiliations:** ^1^ Department of Pathology and Laboratory Medicine Loyola University Chicago Maywood Illinois USA

## Abstract

Urine cytology has long been a challenging diagnostic modality due to its low sensitivity for low‐grade urothelial neoplasms and high interobserver variability. The introduction of The Paris System (TPS) in 2016 marked a pivotal shift towards standardisation, with a primary focus on detecting high‐grade urothelial carcinoma (HGUC). This review evaluates the impact of TPS on diagnostic accuracy, reproducibility, and clinical utility. It also highlights the system's limitations, including issues with nuclear‐to‐cytoplasmic (N/C) ratio estimation, cellular degeneration, and the underrepresentation of HGUC variants. The second edition of TPS (TPS 2.0) addresses many of these concerns, offering refined criteria and visual aids. However, further improvements are needed, particularly in the integration of molecular diagnostics and artificial intelligence.

## Graphical abstract

This review highlights the impact of The Paris System on improving diagnostic accuracy in urine cytology, reducing atypia rates and standardising reporting. It also discusses unresolved challenges and updates introduced in TPS 2.0, including refined criteria and the potential role of artificial intelligence.

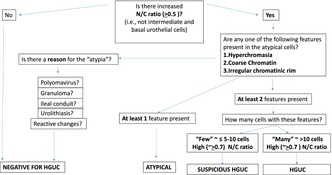

**Abbreviations**
TPS: The Paris System
HGUC: high grade urothelila carcinoma

## Background

Over the years, urine cytology has been one of the most frustrating and challenging areas in diagnostic pathology. Urologists often struggled to understand why urine cytology results were negative despite the presence of papillary tumours. Conversely, surgical pathologists were perplexed when a negative biopsy corresponded with a positive urine cytology report. It seemed that no matter what cytopathologists did, their findings were perceived as incorrect. The results frequently failed to correlate with cystoscopy and biopsy outcomes.

To avoid being consistently wrong, the use of indeterminate categories—such as ‘atypia’—began to rise. At one point, reports indicated that the rate of atypia exceeded 50%.^1^ As a result, clinicians began to view urine cytology as clinically unreliable, equating its usefulness to the flip of a coin. In the end, nearly everyone grew to dislike dealing with urine samples.

Compounding the issue was the absence of standardised diagnostic criteria, which led to significant inter‐ and intra‐observer variability and a lack of reproducibility. Consequently, the credibility of urine cytology steadily declined.^2^

Despite these challenges, the cytology community was not ready to give up. Research groups around the world continued to investigate urine cytology, aiming to identify the key morphologic features critical for diagnosing bladder cancer. Simultaneously, our understanding of bladder cancer pathogenesis improved—particularly the distinctions between low‐grade and high‐grade tumours, especially in terms of clinical presentation, prognosis and significance.

## Creation of the Paris System

The turning point came at the International Congress of Cytology held in Paris in May 2013. During this event, panellists from two Urine Cytology symposia—moderated by Drs. Wojcik and Bubendorf—came together and resolved to address a longstanding issue in the field. This marked the formation of the original Paris group.

At their very first meeting, the group defined the goals and purpose of what would become The Paris System for Reporting Urinary Cytopathology. Most notably, they established that the primary objective of urine cytology is the detection of high‐grade urothelial carcinoma.

In a short span of time, the group expanded to include numerous international experts. These specialists convened on multiple occasions to refine the diagnostic criteria and framework for the urine cytology reporting system.

Their efforts culminated in the 2016 release of a book that introduced diagnostic categories and morphologic criteria grounded in published evidence and data. The first edition of the Paris System (TPS) established a new paradigm: the primary goal of urine cytology is the detection of high‐grade urothelial carcinoma (HGUC).^3^

## Principles of the Paris System

**Figure 1:**
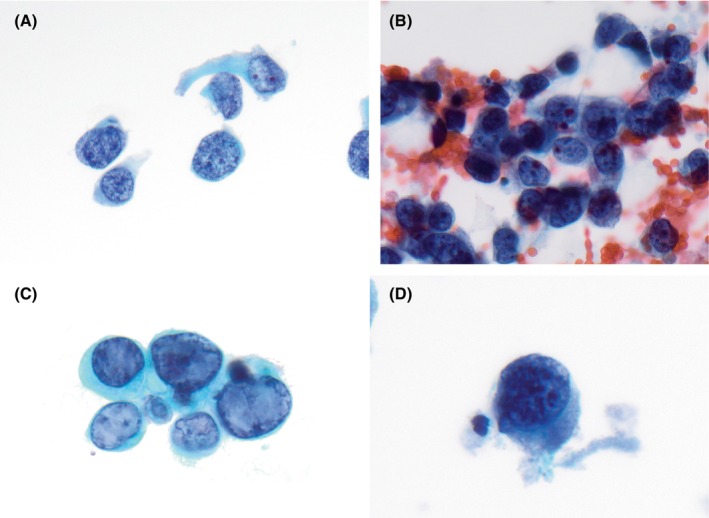
Cytomorphologic features required for diagnosis of HGUC. (**A**) High N/C ratio, (**B**) Hyperchromasia, (**C**) Irregularity of nuclear membrane, (**D**) Coarse chromatin.

Following an extensive review of the literature, key morphologic features predictive of HGUC were identified. These include a high nuclear‐to‐cytoplasmic ratio, hyperchromasia, irregular nuclear membranes and coarse chromatin (Table 1). Based on these features, a confident diagnosis of HGUC can be made (Figure 1).

**Table 1:** Cytomorphologic features required for diagnosis of HGUC (present in at least 5 cells or in at least 10 cells in upper tract).

Cytomorphologic feature	Comments
High N/C ratio (>0.7) (Figure 1A)	Most tumour cells will have a spectrum of N/C ratios
Moderate to severe hyperchromasia (Figure 1B)	Nuclei are dark due to increased DNA content or condensation. Sometimes nuclei are very dark and dense/opaque—‘India ink nuclei’
Irregularity of nuclear membrane (Figure 1C)	Nuclei are often irregularly shaped with coarse and uneven borders
Coarse chromatin (Figure 1D)	Chromatin is clumped and coarse, giving the nucleus a granular appearance

N/C ratio, nuclear/cytoplasmic ratio.

Given this focus, the diagnostic categories were revised to reflect the system's primary aim—detecting high‐grade disease. Categories such as ‘Negative for High‐Grade Urothelial Carcinoma’ and ‘Suspicious for High‐Grade Urothelial Carcinoma’ were introduced.

The working group also demonstrated that low‐grade urothelial tumours cannot be reliably detected by cytology alone, as their morphology closely resembles that of normal or instrumented urine.^4^ The only distinguishing feature is the presence of fibrovascular cores, which indicate a papillary lesion. As a result, fibrovascular cores were established as the hallmark of a new diagnostic category: Low‐Grade Urothelial Neoplasm (LGUN), encompassing papilloma, Papillary Urothelial Neoplasm of Low Malignant Potential (PUNLMP) and low‐grade urothelial carcinoma.

In addition to diagnostic categories, the first edition of TPS included chapters on the pathogenesis of urothelial carcinoma, ancillary studies, clinical management and specimen preparation techniques. Shortly after its release, TPS was translated into Japanese, Russian and Chinese, solidifying its status as an international reporting standard.

## Validation and Evolution of TPS

Despite initial shortcomings, a growing body of literature has confirmed the validity and effectiveness of TPS. Most significantly, the reported global rate of atypia has dropped.^5^, ^6^, ^7^, ^8^, ^9^ Numerous publications have addressed previously unanswered questions and well‐known limitations of the first edition.

One of the most significant studies validating the first morphologic principle of TPS—the nuclear‐to‐cytoplasmic (N/C) ratio—was published by the Johns Hopkins group.^10^ This study confirmed the intuitively selected threshold of 0.5 as a reliable starting point for identifying abnormal urothelial cells. The researchers analysed 200 urine cytology cases categorised as atypical urothelial cells: 100 with negative follow‐up and 100 with positive follow‐up. They evaluated several morphologic features, including N/C ratio, cellularity and nuclear size. Among these, only the N/C ratio effectively differentiated between the two groups. This study validated the 0.5 N/C ratio threshold proposed by TPS as the optimal cut‐off for atypical urothelial cells.

With the accumulation of data based on standardised morphologic criteria, many previously underexplored issues could now be addressed. More importantly, the risk of high‐grade malignancy (ROHM) could be estimated for each diagnostic category.

**Figure 2:**
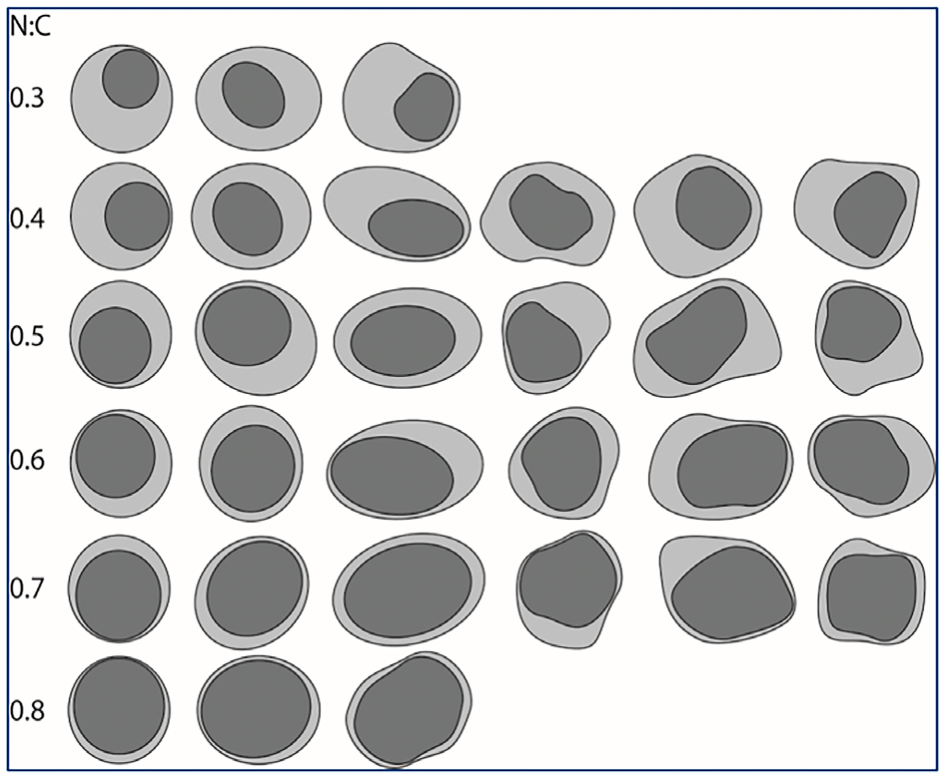
Graphic representations of N/C ratios (from Zhang *et al. Cancer Cytopathol*. 2016;124 (9):669–677).

A few years after the release of the first edition of TPS, sufficient data had been generated to answer numerous previously unresolved questions.^11^ This led to the publication of the second edition.^12^ Given the confirmed significance of the N/C ratio, the second edition included visual aids—graphic representations of specific N/C ratios (Figure 2)—and examples illustrating potential reasons for over‐ or underestimation.

The second edition also attempted to address cellular degeneration, although this issue remains incompletely resolved. In general, cells with incomplete cytoplasm, discontinuous nuclear membranes, and poorly preserved chromatin should be excluded from evaluation for high‐grade urothelial carcinoma or suspicious categories. These cells should instead be classified as atypical urothelial cells.

One of the most notable changes in the second edition was the elimination of the LGUN category as a primary diagnosis. LGUN is now included under the ‘Negative for High‐Grade Urothelial Carcinoma’ category. Fibrovascular cores, previously used to establish LGUN diagnosis, are extremely rarely seen in urine specimens. Moreover, high‐grade urothelial carcinoma can also form fibrovascular cores, potentially leading to underdiagnosis. TPS 2.0 emphasises that diagnosis should be based on cellular features rather than solely on the presence of fibrovascular cores.

The second edition also acknowledges the existence of numerous variants and divergent differentiations of high‐grade urothelial carcinoma. Extensive examples of these variants are included, along with recognition of the hypochromatic variant of high‐grade urothelial carcinoma. The overall changes and alterations of TPS2.0 are summarised in Table 2.

**Table 2:** The modifications and alterations in TPS 2.0

Diagnostic category	Modifications/alterations	Comments
Adequacy	Essentially unchanged Voided urine—25 mL for ThinPrep, 30 mL for SurePath. Instrumented—20 cells/10HPF	Quantitative volume recommendation for voided urine Additional and updated references
NHGUC	LGUN included in this category	LGUN should be a second line diagnosis Further characterisation of degenerative changes
AUC	Essentially unchanged Elevated N/C ratio (between 0.5 and 0.7) and at least one of three minor criteria (hyperchromasia, irregular nuclear membrane and irregular chromatin)	Less emphasis on strict numbers—elevated N/C ratio Elevated threshold for degenerated cells Established expected rate (5%–15%)
SHGUC	Essentially unchanged Few (1–5)cells with high (>0.7) N/C ratio, hyperchromasia and/or irregular nuclear membrane and irregular chromatin	Less emphasis on strict numbers—a few cells and high N/C ratio
HGUC	Essentially unchanged Many (>10) cells with high (>0.7) N/C ratio, hyperchromasia, irregular nuclear membrane and irregular chromatin	Less emphasis on strict numbers—many cells and high N/C ratio. Not all malignant cells will have high N/C ratio Small subset of HGUC has hypochromasia Some variants (HGUC with squamous differentiation, plasmacytoid, micropapillary) will have lower N/C ratio
Non‐urothelial	Previously called other malignancies	ASC a separate entity from AUC

ASC, atypical squamous cells; AUC, atypical urothelial cells; HGUC, high grade urothelial carcinoma; HPF, high power field; LGUN, low grade urothelial neoplasm; N/C ratio, nuclear/cytoplasmic ratio; NHGUC, negative for high grade urothelial carcinoma; SHGUC, suspicious for high grade urothelial carcinoma.

Following the release of the first edition, numerous studies demonstrated that the proposed diagnostic criteria for urothelial carcinoma were effective. The global rate of atypia decreased, and this data was summarised in the second edition. Based on these findings, meaningful and standardised risk of high‐grade malignancy estimates were established for all diagnostic categories. As predicted, the ROHM increased progressively across categories, validating the diagnostic framework.^13^ The summary of the Paris System diagnostic categories, their frequencies and their ROHM are presented in Table 3.

**Table 3:** Paris system diagnostic categories, their frequencies and ROHM

Diagnostic category	Diagnostic category	Frequency	ROHM
Unsatisfactory	Voided urine—volume (>30 mL) Instrumented urine—cellularity	0%–5%	0%–16%
Negative for high grade urothelial carcinoma (NHGUC)	Benign urothelial, glandular, squamous cells, benign tissue fragments, changes due to instrumentation, lithiasis, polyoma virus, therapy. Low Grade Urothelial Neoplasm (LGUN)	70%–90%	8%–24%
Atypical urothelial cells (AUC)	Required—increased N/C ratio (≥0.5) and one of: Hyperchromasia, Irregular clumpy chromatin or Irregular nuclear contours	5%–15%	24%–53%
Suspicious for high grade urothelial carcinoma (SHGUC)	Required—Few cells (<5–10) with high N/C ratio (>0.7) and hyperchromasia, and/or Irregular clumpy chromatin, Irregular nuclear contours	0.5%–3%	59%–94%
Positive for high grade urothelial carcinoma (HGUC)	Required—Many cells (>10) with high N/C ratio (>0.7) and hyperchromasia, Irregular clumpy chromatin, Irregular nuclear contours	0.1%–3%	76%–100%

ROHM, risk of high grade malignancy.

**Figure 3:**
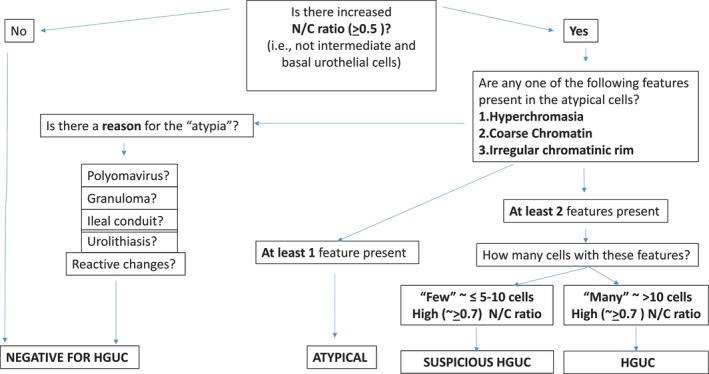
Algorithmic approach to urine cytology specimen based on TPS 2.0.

One common criticism of TPS was the strict adherence to specific numerical thresholds, such as an N/C ratio of 0.7 or the number of cells required to differentiate between ‘Positive for High‐Grade Urothelial Carcinoma’ and ‘Suspicious for HGUC’. The second edition acknowledges case‐to‐case variability and recommends using quantitative descriptors – such as ‘few cells’, ‘numerous cells’, ‘very high N/C ratio’ or ‘increased N/C ratio’ – instead of rigid numerical values. These descriptors correspond to recommended value ranges but allow for greater diagnostic flexibility. Figure 3 represents an algorithmic approach to urine cytology specimen based on TPS 2.0.

## Conclusion

The Paris System for Reporting Urine Cytology has transformed the field of urinary cytopathology by introducing a standardised, evidence‐based framework focused on the detection of high‐grade urothelial carcinoma. Its implementation has led to a measurable reduction in atypia rates, improved diagnostic reproducibility and enhanced clinical relevance. The second edition of TPS has addressed many of the limitations of the original system, including the challenges of N/C ratio estimation, cellular degeneration and the recognition of morphologic variants of HGUC.

Despite these advancements, certain areas still require further refinement. The accurate interpretation of degenerated cells, the integration of ancillary testing, and the incorporation of artificial intelligence tools remain ongoing challenges. Continued research and collaboration within the cytopathology community will be essential to further improve diagnostic accuracy and patient outcomes.

TPS has proven to be a robust and adaptable system that not only standardises reporting but also lays the groundwork for future innovations in urinary cytology. Its evolution reflects the dynamic nature of diagnostic medicine and the commitment of cytopathologists to improving patient care through precision and clarity.

## Funding information

No funding was received in relation to the content presented in this manuscript.

## Conflict of interest

The author serves as an editor of The Paris System for Reporting Urinary Cytology. The data presented has been compiled from previously published sources. The author assumes full responsibility for all aspects of the manuscript's creation and development.
